# Stable atypical chest pain with negative anatomical or functional diagnostic test: Diagnosis no matter what or prevention at any cost?

**DOI:** 10.1002/clc.23250

**Published:** 2019-08-20

**Authors:** Deborah Cosmi, Beatrice Mariottoni, Franco Cosmi

**Affiliations:** ^1^ Department of Cardiology Gubbio and GualdoTadino Hospital Perugia Italy; ^2^ Department of Cardiology Cortona Hospital Arezzo Italy

**Keywords:** anatomical testing, functional stress testing, modifiable lifestyle factor control, stable atypical chest pain

## Abstract

**Background:**

Approximately 1% to 2% of patients with stable atypical chest pain (SACP) experienced a major coronary event, even after a negative functional or anatomical test.

**Methods:**

Over the past 15 years, 1706 patients with SACP evaluated in our clinics underwent functional stress testing or coronary computed tomographic angiography (CTA). In these patients, we also assessed the presence of three major modifiable lifestyle‐related risk factors (cigarette smoking, low intake of fruit and vegetables, and physical inactivity). Patients were stratified according to the presence of at least one risky lifestyle factor or no risky lifestyle factors. Functional or anatomical tests were positive in 170 patients (10%). We followed the remaining 1536 patients with negative tests for 1 year to evaluate the incidence of major coronary events.

**Results:**

The percentage of patients reporting major coronary events was 1.2% in the group with risky lifestyles and 0.2% in the non‐risky lifestyle group (*P* < .01). Events were more common in smokers.

**Conclusions:**

Patients with SACP, when functional or anatomical tests are negative, have a residual risk of fatal and non‐fatal cardiovascular events of 1% at 1 year of follow‐up. People with incorrect lifestyles, especially smokers, have a higher risk of events. We think that in this population, a more effective intervention on lifestyles could be the key to reduce major cardiovascular events.

## 1 BACKGROUND

In general, approximately 2% of the consultations required by patients are related to chest pain.^1^ In most cases, this symptom is not due to cardiovascular causes. However, coronary heart disease is the first diagnosis to be excluded because it is life‐threatening. A cardiologic evaluation is often required in the emergency department (ED) in cases of clinical instability or outpatient instability when the clinical picture is stable. At the outpatient level, in 5.5 years of follow‐up, 4.7% of patients without a precise diagnosis of chest pain and 3% of those in whom a coronary artery disease was excluded experienced a cardiovascular event.^2^ In recent years, it has been shown that more than one‐third of symptomatic patients have a non‐obstructive coronary artery disease (ischemia and no obstructive coronary artery disease, INOCA) without a fully benign prognosis.^3^, ^4^

A recent meta‐analysis of 13 controlled studies about patients evaluated in the ED or doctor's office, performed between 2007 and 2016, found a substantial equivalence in terms of cardiovascular events between functional (exercise electrocardiography, stress echocardiography, and nuclear stress testing) and anatomical testing (coronary computed tomographic angiography, CTA), with an 18‐month follow‐up mortality rate of 1.1% for functional tests and 1% for CTA.^5^ The rate of hospitalization was similar (2.7% vs 2.7%). In patients evaluated with CTA, there was a lower incidence of myocardial infarction (0.7% vs 1.1%) with the need to perform 250 TC in 18 months to avoid a heart attack. During a mean follow‐up of 18 months, all‐cause mortality and cardiac hospitalizations were similar in the CTA and functional testing groups, whereas myocardial infarction (MI) was significantly less common among CTA patients. However, CTA was associated with significantly higher rates of invasive angiograms (12% vs 9%), revascularizations (7% vs 4%), and medical therapy with aspirin or statins (roughly 20% vs 8%). Even in controlled trials that only evaluated outpatients (Min et al. PROMISE, SCOT‐HEART, CAPP),^6^, ^7^, ^8^, ^9^ there were no significant differences between the various tests, with a relatively low (1%‐2%/year) incidence of major clinical events (death and non‐fatal MI). In the PROMISE study, approximately 1% of patients with negative functional or anatomical tests experienced a cardiovascular event.^7^ The anatomical evaluation allows a better diagnosis of non‐obstructive coronary artery disease but not a significant reduction of fatal and non‐fatal events compared to the functional evaluation.^10^ In the 5‐year SCOT‐HEART extension study, anatomical investigations with coronary CT scans was superior to the exercise electrocardiogram (ECG),^11^ resulting in a lower incidence of non‐fatal heart attacks but not of mortality. Even the evaluation of parameters such as the ankle‐brachial index (ABI), high‐sensitivity C‐reactive protein (hsCRP) level and coronary artery calcium (CAC) score (JAMA) significantly improves the prognosis.^12^

The choice of the test is variable and often influenced more by the cardiologist's preferences and experience rather than by evidence‐based medicine. In addition to the diagnostic analysis, it is important to analyze the patient's lifestyle for potentially modifiable factors. In the analysis of lifestyle factors and risk factors in countries with relatively high sociodemographic levels in the 1990 to 2016 period, cigarette smoking and diet are the main factors affecting both overall and individual cardiovascular risks.^13^ In the PROMISE and SCOT‐HEART trials, smokers accounted for 51% and 53%, respectively, while patients with sedentary lifestyles accounted for 48% in the PROMISE trial and were not evaluated in the SCOT‐HEART trial. In the study by Min et al, smokers accounted for 51%, while they accounted for 47% of those in the CAPP trial. Data on fruit, vegetable and legume consumption were not reported in any of the four studies. In cohort studies, in the presence of non‐risky lifestyle factors, the prognostic improvement is clear, with a reduction in the incidence of ischemic events up to 86%, as highlighted in the INTERHEART Study, HALE Trial, EPIC‐Norfolk study, Swedish Register, PURE Diet, and PURE Activities.^14^, ^15^, ^16^, ^17^, ^18^, ^19^

In our analysis, we assessed the incidence of fatal and non‐fatal ischemic events at 1 year of follow‐up in patients with stable atypical chest pain (SACP) and an intermediate pre‐test probability (PPT) who lacked a history of previous cardiovascular events and had negative functional or anatomical testing. These patients were considered to be at low risk and were stratified for the presence of risky lifestyle factors (smoking; diet poor in fruits, vegetables and legumes; and lack of physical inactivity).

## 2 METHODS

In the last 15 years, 36 244 patients >35 years old were examined in our clinics. Of these, 3588 (10%) reported that they had experienced chest pain for more than 2 days at the date of evaluation (Table 1). We excluded patients with diagnosed or suspected acute coronary syndromes at the time of the evaluation (88 patients, 2.5%), known coronary artery disease (172 patients, 4.7%), and patients with significant congenital defects, significant valve diseases, cardiomyopathies, or known or suspected channelopathies (30 patients, 0.8%). The final assessment involved 3298 patients (9%).

**Table 1:** Reason for a referral to the clinics

Chest pain	**3.588**	**10%**
Dyspnea	2.608	7%
Palpitations	2.314	6%
Presyncope	1.956	5%
Others	2.174	7%
Follow‐up	13.064	36%
Check up	10.540	29%
Total	36.244	100%

Bold values are focus on patients with chest pain.

The cardiologic examination and ECG were performed at the request of the general medical practitioner after booking at the Unified Reservation Center (Centro Unico di prenotazione (CUP)), with a median waiting time of 10 days. Patients requiring an urgent cardiologic examination were excluded from the analysis. At the time of the visit, chest pain was defined according to the Diamond and Forrester classification as typical angina (typical retrosternal chest pain of typical type and duration, caused by exercise or emotional stress, relieved by rest and/or nitrates within minutes), atypical chest pain (meeting two of the previous characteristics), and non‐anginal chest pain (satisfies only one or none of the above characteristics) (Table 2).^20^ In cases of non‐anginal chest pain, a stress test was not recommended. Patients with typical chest pain underwent an exercise ECG that was performed within 1 to 3 days of the visit at the discretion of the cardiologist. The PPT of stable coronary artery disease was also calculated based on the ESC guidelines.^20^

**Table 2:** Chest pain characteristics in patients without previous cardiovascular events

	Women	Men	Total
Typical chest pain	128 (7%)	244 (16%)	372 (11%)
Atypical chest pain	906 (50%)	800 (54%)	1706 (52%)
Non‐anginal chest pain	774 (43%)	446 (30%)	1.220 (37%)
Total	1.808	1490	3.298

In cases of atypical chest pain, the same stress test was booked through the CUP. The median waiting time for testing was 20 days. If the exercise ECG was negative or not feasible due to the lack of an evaluable ECG or the patient's inability to perform the test, an imaging test (scintigraphy, eco stress, and coronary CTA) was also proposed. CTA was performed with the use of a 64‐slice or greater multidetector CT scanner. The stress test was considered to be positive according to the American Heart Association guidelines.^21^ Patients with a pre‐test likelihood of 15% to 85% were stratified for the presence of lifestyle‐related risk factors (smoking; low fruit, vegetable, and legume intake; physical inactivity) at the time of enrolment according to their answers on a specific questionnaire (defined as 0‐5‐30; 0 = zero cigarettes, 5 = 5 portions of fruit, vegetables and legumes, 30 = at least 30 minutes of moderate physical activity per day). All patients underwent healthy lifestyle counseling. After 12 months (with a tolerance of ± 2 weeks), a final visit was performed. The presence of major risk factors necessitating pharmacological treatment (hypertension, dyslipidemia, and diabetes) and family history of ischemic heart disease was also noted.

## 3 RESULTS

Table 2 shows the distribution of patients based on the characteristics of the pain. The pain was non‐anginal in 37% of cases (43% of women, 30% of men), atypical in 52% of cases, and typical in 11% of cases. No functional or anatomical tests were performed in patients with non‐anginal pain, and no cardiovascular events occurred in 1 year of follow‐up in this group. In all 372 patients with typical chest pain, coronary angiography was performed, and revascularization was performed in 82% of cases. In 1706 patients with atypical chest pain, the PPT was calculated, and functional or anatomical tests were performed for the detection of possible ischemia. The test was positive in 6% of patients with PPT 15% to 65% and in 20% of those with PPT 66% to 85% (altogether in 170 patients, 10%) (Table 3). These patients underwent coronary angiography. Sixteen patients with atypical but recurrent chest pain and negative functional tests also underwent coronary angiography. In 14 patients, it was negative, and in two patients, non‐obstructive coronary heart disease was identified. Table 4 shows the main basal characteristics of the 1636 patients with negative functional or anatomical testing, stratified for at least one risky lifestyle factor. A total of 27% of patients had non‐risky lifestyles, while 73% had one or more risky lifestyles factors. At the 1‐year follow‐up, 1% (9 males, 6 females) of the patients had experienced a coronary event (four sudden deaths, three non‐fatal ST elevation myocardial infarctions (STEMIs) and eight non‐fatal non ST elevation myocardial infarction (NSTEMIs)) (Table 4). These events were present in 0.8% of the 764 patients with PPT 15 to 65, 2.2% of the 354 patients with PPT 66 to 85 and at least one risky lifestyle factor (overall 1.2%) and one (0.2%) of the 418 patients with PPT 15 to 85 without risky lifestyle factors (*P* < .01). Of the 15 patients with coronary events, 11 (73%) were smokers. The four patients who experienced sudden death (0.3%) were all heavy smokers (>20 cigarettes/day). None of the patients with cardiovascular events had changed their lifestyles during the follow‐up. Coronary events occurred at a median time of 5 months from the baseline evaluation (Table 5).

**Table 3:** Tests results in patients with atypical chest pain

	No. of patients	Exercise ECG	Nuclear stress testing	Stress echo	CTA	Positivity ≥ 1 test
PPT 15‐65	1.146	886	500	116	36	64 (6%)
PPT 65‐85	560	444	300	50	14	106 (20%)
Total	1.706	1.330 (78%)	800 (46%)	166 (10%)	50 (3%)	170 (10%)

*Note*: Some patients underwent more than one test.

Abbreviations: CTA, computed tomographic angiography; ECG, electrocardiogram; PPT, pre‐test probability.

**Table 4:** Characteristics of patients with atypical chest pain and negative tests

	Adequate life‐styles (418) (27%)	Inadequate life‐styles (1.118) (73%)
Age	60.1 ± 9.1	60.9 ± 8.9
Women	107 (51%)	280 (50%)
Hypertension on treatment	11%	14%
Diabetes on treatment	4%	6%
Dyslipidemia on treatment	8%	7%
Family history	2%	2%
Smoking	—	44%
No physicalactivity	—	41%
Diet: <5 portions of frui‐vegetables/day	—	68%

**Table 5:** Major events (sudden death or myocardial infarction) at 1 year follow‐up in patients with stable atypical chest pain and negative functional or anatomical tests

Pre‐test likelihood	Inadequate life‐styles no. of patients	Events	Adequate life‐styles no. of patients	Events	*P*
Pre‐test probability (PPT) 15‐65	764	6 (0.8%) (5 smokers)	338	0 (0%)	<.01
PPT 66‐85	354	8 (2.2%) (6 smokers)	80	1 (1.2%)	<.01
Total	1.118	14 (1.2%) 4 (0.3%) fatal	418	1 (0.2%) 0 (0% fatal)	<.01

## 4 DISCUSSION

Patients with stable, atypical pain and an intermediate PPT are a challenge for clinicians. In these patients, there is a residual risk of about 1% of fatal and non‐fatal events, which can hardly be reduced even with the most sophisticated diagnostic tests.

These patients represent a high risk for the physicians, due to the important medical‐legal implications that can follow when an event occurs. The doctor has two choices: (a) to get involved in very expensive and often useless diagnostic procedures since they do not modify the patient's prognosis; (b) Try to modify the risk factors and above all the lifestyles, with a clear improvement of the prognosis, as we demonstrated also in our study, in which the events were of 1.2% in patients with risky lifestyles and 0.2% in those with correct lifestyles. Especially, heavy smokers with negative diagnostic tests have a high incidence of events, many of which are fatal.

The positivity rate of functional or anatomical tests in these patients is low (10% in our study, 11% in the PROMISE and SCOT‐HEART trials). Patients with atypical pain and a positive test for ischemia may have a prognostic improvement after revascularization if the symptoms persist despite optimal medical therapy, coronary stenosis >80% or 40% to 80% is found, and the instantaneous wave‐free ratio (IF) is ≤0.89.^22^ Patients without these characteristics can improve their quality of life with revascularization if their symptoms are very disabling, but the prognosis quoad vitam is not improved compared to those treated with only optimal medical therapy. Patients with atypical chest pain and negative functional testing are considered at low risk. In these cases, CT can identify non‐obstructive coronary artery disease, which has a worse prognosis than the absence of coronary artery disease. These patients have a likelihood of events within a very wide range (between 0.1% and 1.9%). In those with the non‐obstructive involvement of three vessels, the risk of fatal and non‐fatal events is >3% per year.^23^ Sometimes, in these patients, there is a real “therapeutic obstinacy” that does not necessarily lead to a better prognosis. Overall, the risk‐benefit and cost‐benefit ratios of nuclear tests, cardiac magnetic resonance and CTA are unclear because of the possibility of overdiagnosis and overtreatment, especially with regard to inappropriate revascularization. Methods of reducing the residual risk in these patients remain challenging. It has recently been found that in the general population, high cardiac troponin concentrations within the normal range are associated with a higher incidence of cardiovascular events.^24^ Whether this can improve the prognostic prediction in these patients remains to be established.

The PPT considered in the ESC guidelines takes into account the characteristics of the patient's pain, age, and sex.^20^ It does not take into account lifestyle factors that strongly affect the prognosis of these patients. In the INTERHEARTcase study, the presence of non‐risky lifestyles, such as the absence of a smoking habit of any form; adequate daily intake of fruit, vegetables, and legumes; and sufficient physical activity, leads to an 80% reduction in the risk of infarction.^14^ In total, 65% of reduction is attributable to smoking cessation; 30% to proper fruit, vegetable, and legume consumption; and 14% to adequate physical activity. Similar results were found in other studies, such as the EPIC‐Norfolk trial, Swedish Registry, HALE trial, PURE diet, and PURE activities.^15^, ^16^, ^17^, ^18^, ^19^ In our study, we have shown that patients with atypical stable chest pain and negative functional diagnostic tests with non‐risky lifestyles have a significantly better prognosis than patients with risky lifestyle factors (0.2% of patients with non‐risky lifestyles with events at the 1‐year follow‐up compared to 1.2% of patients with at least one risky lifestyle factor, *P* < .01). In the real world, however, consideration should be given to the fact that changing lifestyle factors is very difficult in practice and resisted by patients (recently it has been termed Sisyphus fatigue).^25^ In particular, cigarette smoking is associated with a very high risk of sudden death, so much so that in the US guidelines, cigarette smoking abstention is placed first in the recommendations for preventing this mode of death.^26^ Worldwide, 25% of men and 5% of women are smokers (in Italy, 23% and 17%, respectively).^24^ In our study, the majority of patients who experienced events were current smokers. Even in those with diagnosed (revascularized or non‐revascularized) coronary artery disease, the persistence of smoking habits is the most important lifestyle‐related risk factor for future coronary events and is associated with a lower efficacy of statin therapy.^27^

These patients should be initiated on pathways to improve their lifestyles and especially on pathways that favor the cessation of cigarette smoking. In a systematic review of the scientific literature on lifestyle changes in patients with chronic ischemic heart disease, it has been found that the cessation of smoking, introduction of a healthier diet and engagement in physical activity may lead to a 36%, 44%, and 24% reduction in mortality, respectively.^28^ Recently, it has been found that in obese patients without metabolic alterations, the incidence of coronary heart disease is 49% higher than in non‐obese patients.^29^ Unfortunately, lifestyle modification is a challenging task. In the EUROASPIRE V trial, over 1 year after the diagnosis of acute coronary syndrome, 19% of smokers continued to smoke, 66% did not engage in any physical activity, 44% were overweight and 38% were obese.^30^

Even in the COURAGE study, after 5 years, the proportion of patients who smoked was only reduced from 23% to 19%, the proportion of patients who engaged in physical activity increased from 58% to 66% and body mass index (BMI) increased from 28.8% to 29.3 kg/m^2.^ 31 The best results were obtained in the recent RESPONSE‐2 study, in which intervention led to a significant improvement in lifestyles (37% in the case group vs 26% in control group),^32^ and in the Nurses' Health Study and Health Professionals Follow‐up Study, in which there was a total mortality reduction of 17%.^33^

Of course, in these patients, the pharmacological treatment of risk factors (low‐density lipoprotein cholesterol, hypertension, blood glucose) should be optimized. In particular, statin treatment reduces the risk of overall mortality by 14% and that of cardiovascular disease by 31%.^34^ However, it should be stressed that if risky lifestyle factors persist (especially cigarette smoking), the residual risk remains high despite treatment with statins.^35^ Revascularization improves the quality of life by reducing symptoms in patients who have recurrent chest pain despite optimal medical therapy but does not reduce either the risk of heart attack or the risk of death.^16^

Our analysis has several limitations. First, it described a single‐center experience; second, the most widely used test was exercise ECG. However, it should be emphasized that we started to collect our data approximately 15 years ago, when CTA was not widely performed in Italy, and that in uncertain cases, we performed more than one test. Exercise ECG has a low sensitivity (45%‐50%), but a good specificity (85%‐90%) and in the SCOT‐HEART trial, it was used in 85% of the patients.^8^

## 5 CONCLUSIONS

Patients requiring an outpatient cardiac evaluation for atypical chest pain, with negative functional tests and/or imaging diagnostics (including CTA), who were therefore considered low risk have a residual risk of 1.0% for cardiovascular events (1.2% of patients with risky lifestyle factors and 0.2% of those without risky lifestyles) and even fatal events (0.3%) after 1 year. The prognosis, irrespective of instrumental data, could be improved by optimizing medical therapy, especially with the correction of major lifestyle‐related risk factors (smoking, poor diet, physical inactivity), which can lead to a reduction in the incidence of MI greater than 80%.

In the real world, however, the correction of lifestyle‐related risk factors is challenging. In these patients, a shared decision‐making between physicians and patients based on demonstrated effectiveness should be initiated, stressing that, if risky lifestyle habits persist, the risk of cardiovascular events at 1 year is quantifiable at approximately 1% if PPT is 15% to65% and 2% if PPT is 66% to 85%, regardless of the outcome of the instrumental exams. A strict control of lifestyle‐related factors as well as an aggressive treatment of risk factors, which should be implemented for the overall prevention of coronary heart disease after the first negative functional or anatomical test, could improve the clinical outcomes of these patients more than expensive, uncomfortable, and probably futile diagnostic testing. These results need to be confirmed by studying a wider population, and interactions between lifestyles and traditional risk factors such as hypertension, dyslipidemia, hyperglycemia, and familial history of ischemic heart disease remain to be established.

## CONFLICT OF INTEREST

The authors declare no potential conflict of interests.

## AUTHOR CONTRIBUTION

Deborah Cosmi and Franco Cosmi contributed to the conception of the work. Deborah Cosmi, Beatrice Mariottoni, and Franco Cosmi contributed to the acquisition, analysis, or interpretation of data for the work. Deborah Cosmi and Franco Cosmi drafted the manuscript. All authors critically revised the manuscript, gave final approval and agree to be accountable for all aspects of work ensuring integrity and accuracy.
